# Barriers and facilitators for oral health screening among tobacco users: a mixed-methods study

**DOI:** 10.1186/s12903-024-04084-1

**Published:** 2024-03-05

**Authors:** Abdullah Alsoghier, Abdulrahman Alnutaifi, Obaid Alotaibi, Abdulaziz Alotaibi, Abdullah Alharbi, Nada Almubarak, Sara Albassam

**Affiliations:** 1 Department of Oral Medicine and Diagnostic Sciences, College of Dentistry, King Saud University, Riyadh, 12372 Saudi Arabia; 2 Dental Internship Training Programme, College of Dentistry, King Saud University, Riyadh, Saudi Arabia; 3 Smoking Cessation Clinic, Counselling and Guidance Center, Deanship of Student Affairs, King Saud University, Riyadh, Saudi Arabia; 4 College of Humanities and Social Sciences, Social Studies Department, King Saud University, Riyadh, Saudi Arabia; 5 Department of Basic Dental Sciences, College of Dentistry, Princess Nourah bint Abdulrahman University, Riyadh, Saudi Arabia

**Keywords:** Health services accessibility, Tobacco use, Tobacco use cessation, Health promotion, Oral health, Mouth diseases

## Abstract

**Objectives:**

Tobacco consumption adversely affects general and oral health and is considered one of the significant public health burdens globally. The present study aims to assess the barriers and facilitators for attending oral and dental health screening among tobacco users who seek cessation advice.

**Methodology:**

The present mixed-methods study used group concept mapping (GCM) to identify the facilitators/barriers to attending oral health screening among young adults attending face-to-face and virtual Tobacco Cessation Clinic at King Saud University (Riyadh, Saudi Arabia) between September 2022 and April 2023. Study investigators included healthcare social workers, dental interns, and oral and maxillofacial medicinists. Information about demographics, general health, oral/dental health and tobacco use were collected using self-completed questionnaires. The barriers and facilitators were assessed following GCM by brainstorming, sorting, rating, and interpretation activities. Descriptive, multidimensional scaling and hierarchical cluster analysis were used to describe the study participants and produce concept maps of the generated statements.

**Results:**

The study included 148 participants who generated 67 statements summarised into 28 statements as facilitators or barriers. Based on a 5-point importance scale, the participants indicated the importance of facilitators under health-related cluster [e.g. *when I feel pain*] as the highest, followed by personal [e.g. *to maintain my mouth hygiene*], social [e.g. *the quality of treatment*] and financial clusters [e.g. *the reasonable cost*]. Concerning barriers, financial factors [e.g. *high cost*] acted as the highest-rated barrier, followed by personal [e.g. *lack of dental appointments*] and health-related [e.g. *worry that dental problems will worsen*]. The social factors were the least considerable barrier [e.g. *lack of time*]. Clustering these facilitators/barriers on the concept map indicated their conceptual similarity by an average stress value of 0.23.

**Conclusion:**

Pain was the most important facilitator to attending oral health screening by young adults seeking tobacco cessation advice. Notable barriers included the high cost of dental treatment and the lack of scheduled appointments. Thus, oral health care providers need to consider scheduling periodic and timely dental check-ups to prevent and reduce the burden of tobacco-associated and pain-causing oral diseases.

**Supplementary Information:**

The online version contains supplementary material available at 10.1186/s12903-024-04084-1.

## Background

Tobacco use is a public health burden worldwide that adversely affects general and oral health [1]. It contributes to an annual global mortality rate of approximately 8 million people [2]. The estimated number of tobacco users increased from around 0.99 billion in 1990 to 1.14 billion individuals by 2019 and contributed to direct and indirect economic burdens of $1.8 trillion [3]. Despite the anti-tobacco policies and campaigns in Saudi Arabia [4], tobacco use has contributed to around 70,000 deaths annually and a 9% increase in years lived with disability between 1990 and 2017 [5, 6]. Moreover, tobacco use has collectively contributed to direct and indirect economic burdens of $20 billion in Saudi Arabia [7, 8].

Regular tobacco consumers (e.g. chewed or smoked) are more likely to experience dental fear and dental visit avoidance than those who occasionally or never used tobacco [9]. This feeling may act as a barrier to seeking oral and dental health screening necessary to reduce the risk of tobacco use-associated oral diseases such as dental caries, periodontal disease, fungal infections, potentially malignant oral cavity disorders, and oropharyngeal cancer [10]. Head and neck cancers, including oral squamous cell carcinoma (OSCC), are the sixth most common type of cancer globally and often present a poor 5-year survival rate of 50% and mainly result from smoking [11].

Smoking cessation, in turn, can lead to a 50% reduction in oral and oesophageal cancer incidence within 5 years of quitting [12]. Similarly, males who stopped before age 65 can obtain an estimated average of 2 additional years of life, while females can gain up to 3 years [13]. Another risk reduction strategy of smoking cessation consultation is the early detection of malignant and premalignant changes in the oral mucosa [14, 15]. Indeed, it was found that triennial oral cancer screening of high-risk populations significantly reduced the mortality rate by 27% compared to no screening over a 9-year follow-up period [16]. High-risk individuals may encounter barriers to attending the screening for their oral and dental health, such as limited patient-clinician discussions and insufficient knowledge about cancer risk, lack of routine dental appointments, the cost considerations (e.g. access, treatment and transportation), and dental fear/avoidance behaviour [17–19]. Addressing the psycho-socioeconomic constructs underpinning these barriers is necessary to implement evidence-based anti-tobacco interventions, specifically within the dental practice [18, 20].

The Health Belief Model (HBM) offers insights into health behaviour change concerning tobacco use by considering individuals’ health perceptions and self-efficacy [21]. This model identifies various factors that influence tobacco cessation, including symptom perception (e.g. being diagnosed with oral or dental disease), social influence (e.g. receiving advice from medical or dental practitioners), and health education campaigns (e.g. smoking cessation awareness campaigns) [22]. In the context of head and neck cancer prevention, the HBM can also predict smoking cessation intention by considering perceived susceptibility, seriousness, benefits, barriers, cues to act, and self-efficacy [23].

Group concept mapping methodology (GCM) is a mixed-methods participatory approach that combines group processes, such as brainstorming, sorting, rating, and interpretation, with statistical analysis steps [24, 25]. This method was suitable to identify the factors perceived by employees with rheumatoid arthritis as necessary for preventing work disability and understanding why knee and hip osteoarthritis patients choose specific types of treatment [26–28]. Moreover, it has been used to highlight priorities and barriers to participation in day-to-day living activities among individuals with Sjogren’s syndrome [25].

There remains little known about the factors that could support or deter attending dental visits and specifically oral cancer examinations by tobacco users, especially in the Middle East, North Africa and Saudi Arabia [29, 30]. Identifying the barriers and facilitators could support evidence-based healthcare policy-making for the proportionally increased number of individuals who use tobacco in the region [30, 31]. Therefore, the present mixed-methods study aimed to identify the barriers and facilitators to attending oral and dental health screening among young adults who use tobacco (smoked or chewed) and seek tobacco cessation advice.

## Methods

Following the GCM methodology [24], the prospective single-centre study was conducted from September 2022 to April 2023 at King Saud University’s clinic in Riyadh, Saudi Arabia. The tobacco cessation service of this clinic offers onsite and virtual consultations by walk-in or electronic form-based requests. Those who opted for the virtual clinics were contacted on their chosen date, time, and communication method (telephone or video conference). This clinic, annually attended by more than 1500 university students, staff and the public, was established in 2005 in line with the national endorsement of the WHO Framework Convention on Tobacco Control, aiming to provide counselling and interventional services for those who intend to quit smoking and support their health literacy toward health effects of the tobacco use and exposure [32, 33].

The non-random convenience sampling method was used to invite individuals aged 18 or above who used any form of tobacco and attended the clinic to participate in the study [34]. Those who agreed were asked to read and sign the informed consent form, complete the study forms, and participate in the study phases online or face-to-face based on their preferences. No sample size calculation was needed as recruitment continued until there were no distinctive insights during the GCM-based study activities [24, 35], and data was considered saturated by the study team concerning the facilitators and barriers (21-22).

**Fig. 1:**
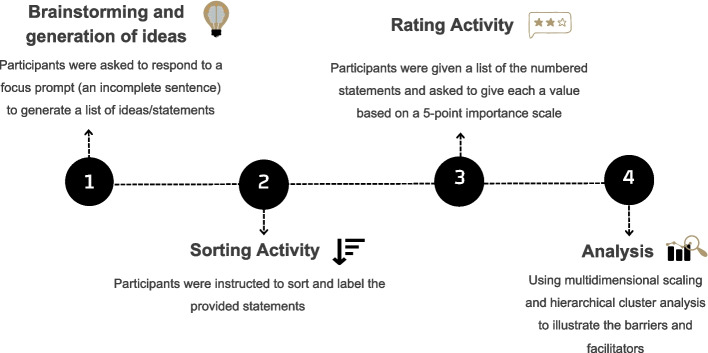
The study phases to identify and illustrate barriers and facilitators for oral health screening among tobacco users

The self-completed study forms by participants included questions about demographics (age, gender, marital status, area of residency and educational level), general health (present medical conditions, medications, and allergies), oral/dental health (oral hygiene habits, frequency of dental visits) and tobacco use (type and frequency) (Supplementary File 1). The study phases needed to identify and represent the facilitators and barriers to attending oral health screening among tobacco users are demonstrated in Fig. 1.

In the initial brainstorming activity [phase 1], the research team (OA, AN and AO) asked each participant independently to consider two statements (in the Arabic language): “*What motivates and helps me to visit the dentist for an oral health check is …”* [facilitators] and *“I do not visit the dentist for an oral health check because…”* [barriers] (Supplementary File 2). The duplicate statements were then eliminated and reviewed for syntax and clarity. The participants were then asked to organise cards containing the generated statements into piles of similar statements and give each a label [phase 2]. Furthermore, the participants were given a link with the sorted statements and an option to rank each of them as suggested based on their perceived importance on a 5-point scale (5 = extremely important, 1 = unimportant) [phase 3] [24, 25, 36]. For data analysis [phase 4], multidimensional scaling and hierarchical cluster analysis were used to produce a two-dimensional representation of the statements and to group the statements into clusters based on Ward’s method [25]. This scaling analysis yielded a stress value that was used as a distance metric to assess the adequacy of fit of data within the generated map with an acceptable cut-off level for concept mapping studies at or above 0.36 [37]. These clusters were then visualised to form a map demonstrating how these points are connected [38].

As the data generated by the study participants was in their native language (Arabic), the forward-backwards translation (Arabic-English-Arabic) was performed by the study team to ensure no discrepancy between the original and translated data for analyses [39]. The data was represented using Microsoft Excel (v. 16.73) and exported to IBM SPSS statistics software (v. 28.0) and MAXQDA (v. 2022) for descriptive statistics and multidimensional scaling/hierarchical cluster analysis, respectively [40].

## Results

The study included 148 participants with a mean age of 29.5 years and a range between 20 and 48 years at the time of recruitment. Of note, 93.9% of the participants were males (*n* = 139), and only 6.1% were females (*n* = 9). The demographic characteristics indicated that 56% of participants were single (*n* = 83), and 77% had a bachelor’s degree or higher (*n* = 115). Also, 131 of the 148 participants (88%) indicated no current medical condition. Regarding oral health behaviours,67% indicated regular use of toothbrush at least once daily, 62% use of dental floss, and 22% noted regular dental visits (Table 1).

**Table 1:** The background and health information of the study population (*n* = 148)

Variable	Category	No of participants (%)
Background		
Gender	Male Female	139 (94%) 9 (6%)
Age	20-35 years 36-48 years	114 (77%) 34 (23%)
Martial status	Single Married	83 (56%) 65 (44%)
Education	College education or higher High school diploma	115 (78%) 33 (22%)
	Current Past	113 (76%) 35 (24%)
General Health and Tobacco Use		
Medical conditions Smoking Type of Tobacco	Yes No Current Past Cigarettes Smokeless Electronic cigarretes Hokah or similar	17 (11%) 131 (89%) 113 (76%) 35 (24%) 109 (46%) 42 (18%) 75 (31%) 12 (5%)
Oral health maintenance		
Toothbrushing Dental floss Regular dental visits	Once daily Twice a day None Yes No Yes No	67 (45%) 32 (21%) 49 (33%) 56 (38%) 92 (62%) 39 (22%) 109 (78%)

During the initial brainstorming activity, 29 participants indicated 67 statements following the prompting questions regarding facilitators and barriers for dental visits, which were further summarised into 28 statements by the research team [OA, AN and AO] (Supplementary File 3). These participants noted feeling pain (*n* = 6), keeping the mouth clean (n = 6) and recognising a problem in the mouth (*n* = 4) as the highest facilitators to seek a dental visit. Highly perceived barriers indicated by participants included a lack of regular dental appointments (*n* = 7), a lack of time (*n* = 6), and believing dental visits were not needed (*n* = 4) (Table 2).

**Table 2:** The summarised list of facilitators and barriers to attending oral health screening in the brainstorming activity (*n* = 29)

**Facilitators**	**No of participants (%)**
“What motivates and helps me to visit the dentist for an oral health check is….”	
*When I feel pain*	6 (%20)
*To keep my teeth clean*	6 (%20)
*When I recognise a problem in my teeth*	4 (%13)
*Ease of dental visit arrangement*	3 (%10)
*To maintain good general health*	3 (%10)
*When plaque build-up*	2 (%6)
*To remove my teeth stains*	2 (%6)
*The quality of treatment*	2 (%6)
*The reasonable cost*	1 (%3)
*To maintain mouth hygiene and prevent bad breath*	1 (%3)
*When I know a good dentist*	1 (%3)
*When I get advice from others*	1 (%3)
*The availability of transportation/easy access to the clinic*	1 (%3)
*The beauty of how my teeth look compared to other people*	1 (%3)
*The discount on screening cost*	1 (%3)
**Barriers**	**Frequency (Percentage)**
“I do not visit the dentist for an oral health check because…”	
*The lack of dental appointments*	7 (%24)
*The lack of time*	6 (%20)
*Dental visits are not needed*	4 (%13)
*The lack of interest*	3 (%10)
*The worry that dental problems will worsen*	3 (%10)
*The long waiting time in the clinic*	2 (%6)
*High cost*	1 (%3)
*The loss of hope in the treatment*	1 (%3)
*The embarrassment from the doctor due to my teeth condition*	1 (%3)
*The fear of pain*	1 (%3)
*No specific reason*	1 (%3)
*Cleaning your teeth twice a day is enough*	1 (%3)
*Not knowing a good dentist*	1 (%3)

The sorting activity included another 17 participants, who listed the 29 generated facilitators/barriers statements under financial, health-related, personal and social clusters. Further to this, another 102 participants rated the importance of previous statements and demonstrated an average importance rate of 4 out of 5 or higher for facilitating statements considered health-related [4.5], personal [4.3], social [4.2] and financial [4] (Table 3). Examples of important statements included feeling pain [4.7], maintaining mouth hygiene and preventing bad breath [4.6], the quality of dental treatment [4.6] and knowing a good dentist [4.5]. In contrast, the participants indicated low importance toward receiving advice from others [3.6], removing teeth staining [3, 7], transportation/access to the dental clinic and discounted screening costs [3.8].

**Table 3:** The importance ratings of the clusters and coded statements in the sorting activity (*n* = 102)

Facilitators	Importance rating
**Health-related Cluster**	**4.5**
F1. When I feel pain	4.7
F4. To keep my teeth clean	4.6
F2. When plaque build-up	4.2
**Personal Cluster**	**4.3**
F5. To maintain my mouth hygiene and prevent bad breath	4.6
F6. When I recognise a problem in my teeth	4.5
F11. To maintain good general health	4.4
F10. To remove my teeth stains	3.7
**Social Cluster**	**4.2**
F12. The quality of dental treatment	4.6
F7. When I know a good dentist	4.5
F14. The beauty of how my teeth look compared to other people	4.2
F8. When I get advice from others	3.6
**Financial Cluster**	**4**
F3. The reasonable cost	4.2
F9. Ease of dental visit arrangement	4.2
F13. The availability of transportation/easy access to the clinic	3.8
F15. The discount on screening cost	3.8
**Barriers**	
**Financial Cluster**	**3.7**
B2. High cost	4.1
B3. Dental visits are not required	3.3
**Personal Cluster**	**3.4**
B1. The lack of dental appointments	4
B5. The long waiting time in the clinic	3.9
B10. The fear of pain	3.5
B7. The lack of interest	3
B11. No specific reason	2.8
**Health-related Cluster**	**3.2**
B9. The worry that dental problems will worsen	3.6
B12. Cleaning your teeth twice a day is enough	3.1
B6. The loss of hope in the treatment	2.7
**Social Cluster**	**3**
B4. The lack of time	3.6
B13. Not knowing a good dentist	2.7
B8. The embarrassment from the doctor due to my teeth condition	2.7

Regarding the barriers, the items under the financial cluster received the highest average rating [3.7], followed by personal and health-related clusters [3.4 and 3.2, respectively]. Within these clusters, barriers with notable significance included high cost [4.4], lack of regular dental appointments [4], long waiting time in the clinic [3.9] and fear of worsening dental problems [3.6]. The lowest rated barriers were however the loss of hope in treatment, not knowing a good dentist, and embarrassment due to one’s teeth condition [2.7 each].

**Fig. 2:**
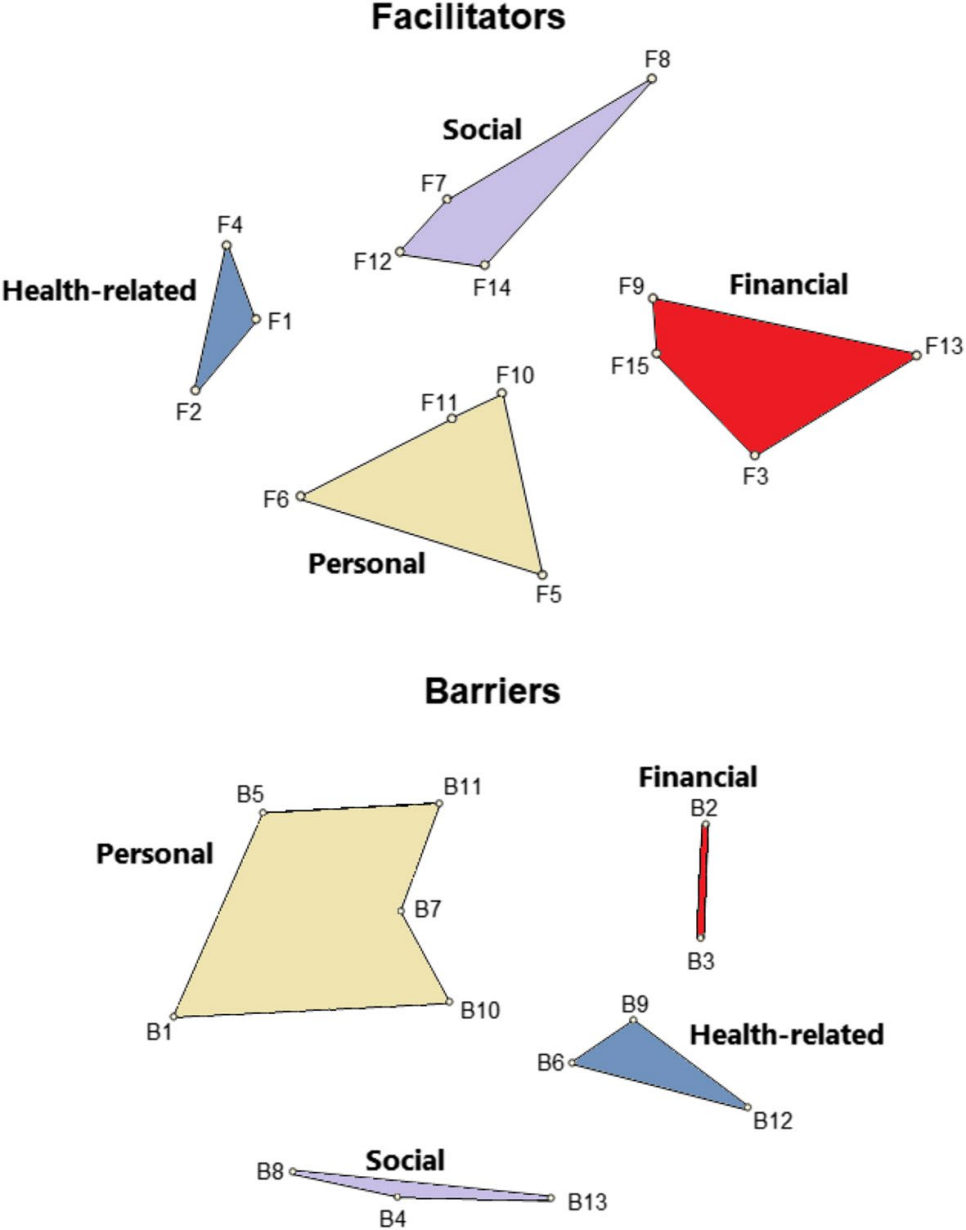
Point cluster map showing the themed clusters of barriers and facilitators for oral health screening among tobacco users

The generated maps confirmed the conceptual relationship between the statements with an average stress value of 0.23, which indicated a goodness-of-fit and stability of statements [facilitators = 0.25, barriers = 0.22] (Fig. 2) [37]. Also, the area formed by statements under each themed cluster in the 2-dimensional polygons (Fig. 2) demonstrated how these statements were conceptually similar or dissimilar to each other [35].

## Discussion

The present mixed-method study followed GCM to explore what could impede a young adult who used tobacco to seek a dental care visit. Encouraging such visits could help early detection and possibly reduce the risk of dental and oral diseases (e.g., oral precancerous changes) and also for counselling and emphasis to quit tobacco use [11, 41]. There remain no regional or KSA-based studies assessing barriers and facilitators to screening among individuals with an increased risk of oral cancer. Previous research has instead focused on access-related barriers among individuals with disabilities [42], older adults [43], the general population [44], and utilising dental services [45].

Similar to the present findings, the determinants of high-risk individuals seeking/accessing dental care visits for oral diseases included personal factors such as dental fear, the lack of motivation/need, and negative experiences [19, 46]. The latter could contribute to dental anxiety and fear often associated with dental visit avoidance and embarrassment [47, 48], which was likely experienced by 2 participants who considered both ‘*fearing that my dental problems will worsen’* [P 15 and 16] and *‘…embarrassment from the doctor due to my dental condition*’ [P 15] as barriers. Also, the participants likely encountered the cost of dental screening and treatment as notable barriers that often hinder seeking care visits for individuals with increased risk of oral diseases and impede their help-seeking behaviour toward suspicious symptoms (e.g. persistent oral mucosal patch or lump), leading to their irregular screening visits [19, 46, 49].

With present participants indicating the high importance of encountering time limit as a barrier (4 out of 5), this factor was previously considered a significant predictor for scheduling and attending oral cancer screening by this population compared to other factors, including the efficacy of coping resources, transportation, and dental exams [50]. Similarly, the participants experienced the reported barriers concerning healthcare services, such as difficulty scheduling appointments and lengthy waiting times at dental clinics [51]. Moreover, the relatively high importance scores given to barriers such as *‘the long waiting time in the clinic’* may reflect an increased dental anxiety level while in the waiting area [52]. It might be notable that these individuals may want to attend an oral health examination but not necessarily intend to ‘adopt’ an action (e.g., scheduling an appointment). This ‘adoption behaviour’ was explained within the health belief model as one’s willingness to engage in making a dental appointment based on weighting the self-perceived benefits (e.g. treating painful tooth) over barriers (e.g. high cost) [23, 50]. Other factors that may affect this adoption behaviour include social norms, cultural beliefs and the fear of being judged or stigmatised due to dental problems [18, 50]. Notably, these factors were highlighted by participants who noticed how their teeth looked, advice from peers and recognised a good dentist as determinants for visiting the dentist.

The present findings confirmed experiencing oral pain as their highest motive to seek dental care, possibly due to poor oral health practices, exposure to tobacco products, reduced saliva volume and changes in the oral microbiome [10, 53]. Indeed, the evidence shows tobacco users are more likely to experience oral health problems and possibly previous pain-associated negative dental experiences [52, 53]. In contrast, quitting tobacco could reduce experiencing oral and orofacial pain symptoms [53] and improve the odds of quitting tobacco, as this pain was a deterrent among 62% of 118 women trying to quit oral smokeless tobacco [54]. It is also worthwhile to consider that there may be other reasons perceived by present or past smokers for not attending an oral screening, such as conflict with work obligations, forgetting the appointment or misunderstanding its purpose, transportation and travel distance [50].

Addressing the present concerns of tobacco users requires interdisciplinary care planning between social care workers and oral healthcare professionals (HCPs) in reducing the highlighted personal, financial, and healthcare service-related barriers to facilitate periodic oral health screening and early detection and management of oral diseases. Social care providers can help understand and navigate the social determinants of health behaviours that may contribute to tobacco use and cessation [55]. These can include low socioeconomic status and deprivation, low levels of education and general/health literacy, inadequate access or transportation issues, and lack of awareness or motivation that could hinder seeking oral health screening [50, 52]. Furthermore, tobacco use is often associated with mental health problems, including anxiety, stress, and social isolation [4, 56]. Therefore, a comprehensive approach by HCPs and policymakers is needed in designing and evaluating cost-effective tobacco-related interventions to reduce the impact on one’s health, social, economic, and psychological aspects [55].

Dental practitioners may consider referring tobacco users to local services in many countries and online sources for further information and advice. For instance, the Florence Artificial Intelligence tool [https://www.who.int/campaigns/Florence] by the World Health Organization (WHO) can virtually provide tailored guidance concerning quitting tobacco use in many languages, including English and Arabic [57]. For instance, the tool answered the question ‘*How can I stop smoking?*’ as ‘*Tobacco is extremely unhealthy and can have a very negative effect on your mental health. Research shows that over half of tobacco users are killed by it. Tobacco kills more than 8 million people each year…Would you like to hear some of the benefits of quitting or hear more about tobacco and mental health?*’ with further suggestions for quitting tobacco. Also, the WHO Collaborating Centre for Smoking Cessation offers online training modules for HCPs and evaluation checklists for the performance and outcomes of such services [58].

The present study used the commonly adopted mixed-method GCM approach to identify the beliefs and attitudes toward seeking oral health screening among tobacco users in a community-based environment. However, the limitations of mixed methods and semi-structured interviews could affect the study findings, such as the Hawthorne effect and the lack of qualitative assessments during sorting and rating activities [50]. To reduce such limitations, the study investigators thoroughly explained the study activities and expectations while assuring anonymous participation [59].

Although there was lower female participation in the study compared to males (9 out of 148), this might parallel the considerably lower prevalence of female tobacco users in Saudi Arabia [60] or those who report its use. The present study also lacks exploring dental professionals’ perspectives on these barriers and facilitators that could help design tailored patient-centred interventions [61]. Unlike other studies, the present analysis lacked assessments concerning socioeconomic factors. Thus, future national and regional studies in the Middle East and North Africa may consider investigating the socioeconomic factors and their impacts on scheduling and attending oral health screening by tobacco users [16, 53]. Also, there is a need to recruit a larger cohort of tobacco users attending tobacco cessation clinical and community-based settings and including the views of their partners and health and social care providers. Examples could include the nationally available Saudi Ministry of Health’s Anti-Smoking Clinics [62] and units allied to national not-for-profit virtual and onsite initiatives to combat tobacco use (e.g. Naqa [63] and Kafa societies [64]).

## Conclusion

The present study indicated that pain was the most important facilitator to attending oral health care services by individuals who consume tobacco and seek tobacco cessation support. In contrast, the high cost of dental treatment and the lack of periodic/scheduled dental reviews were considered significant barriers. Thus, healthcare policymakers and clinicians may consider addressing these barriers for early detection of oral diseases to achieve cost-effective oral healthcare and favourable health-related outcomes for tobacco users.

### Supplementary Information

**Supplementary Material 1.**

**Supplementary Material 2.**

**Supplementary Material 3.**
