# Supplementary material for: Barriers and facilitators for oral health screening among tobacco users: a mixed-methods study
Source: BMC Oral Health. 2024 Mar 5;24:306. doi: 10.1186/s12903-024-04084-1 (PMC10913556; doi:10.1186/s12903-024-04084-1)
Supplement: Supplementary file 1 — Supplementary Material 1. [file 12903_2024_4084_MOESM1_ESM.docx]

Supplementary File 1. The translated and original self-completed background and health information form.

**The background and health information form (translated)**

| **Background Information**  Please complete all of the following questions.   1. Age: __________ 2. Gender:    Male   Female   I would rather not disclose this information   1. Marital status:    Married   Single   Other (please specify):_________   1. City of residence? ___________ 2. Educational level:    Elementary or middle school   High school   University education or higher  **General Health and Tobacco Use Information**   1. Do you have any health conditions currently?    Yes 🡪 If the answer is yes, please indicate the medical condition/s. *_________*   No   1. Do you have any problems with your mouth or teeth at the moment?    Yes 🡪 If the answer is yes, please indicate the problem/s. *_________*   No   1. Tobacco use (for example, regular or electronic cigarettes, hookahs, or chewing tobacco):    I currently use tobacco.   I have used tobacco before.    Please specify one or more types of tobacco used before or currently:   - Chewing tobacco (its type): *_________* - Regular cigarettes (number per day): *_________* - Electronic cigarettes (type and average minutes of daily use): *_________* - Others (type/number per day): *_________*   **Oral health maintenance Information**   1. How many times do you brush and paste your teeth daily?    More than three times a day   Twice a day   Once a day   None of the above     1. Do you use interdental cleaning tools (such as toothpicks, dental floss, or mouthwash)?    Yes   No     1. Do you visit the dental clinic regularly (every 6 months or annually)?    Yes   No 🡪 If the answer is no, when was the last visit to the dental clinic? *_________* |
| --- |

**The background and health information form (original)**

**نموذج المعلومات الشخصية والصحية**

| **المعلومات الشخصية**  الرجاء تعبئة جميع الأسئلة التالية:  ١- العمر: __________  ٢- الجنس:  أنثى  ذكر  أفضل عدم الكشف عن هذه المعلومات  ٣- الحالة الاجتماعية:  أعزب  متزوج  غير ذلك  ٤- مكان الإقامة؟ _______________  ٥- المستوى التعليمي:  المرحلة الابتدائية أو المتوسطة  المرحلة الثانوية  تعليم عالي (جامعي او اعلى)  **معلومات الصحة العامة واستخدام التبغ**  **٦- هل يوجد لديكم أي حالة صحية حاليا؟** لا 🌕 نعم🌕 **إذا الإجابة نعم فما هي**؟ **_________________**  **٧- هل يوجد لديكم أي مشكلة بالفم أو الأسنان حاليا؟** لا 🌕 نعم🌕 **إذا الإجابة نعم فما هي**؟ **_________________**  **٨- استخدام التبغ (مثلا السيجارة الاعتيادية أو الإلكترونية، الشيشة والجراك أو التبغ الممضوغ):** أستخدم التبغ حاليا 🌕 سبق لي استخدام التبغ🌕 **يرجى تحديد نوع واحد او أكثر من التبغ الذي تم استخدامه قبل او حاليا**:  التبغ الممضوغ (نوعه): _____________  السجائر الاعتيادية (العدد يوميا): _____________  السجائر إلكترونية (نوعها ومتوسط دقائق الاستخدام اليومي): _____________  أخرى (النوع/العدد يوميا): __________  **أسئلة لعادات صحة الفم:**  **٩- كم مرة تستخدم الفرشاة والمعجون لأسنانك يوميا**؟  🌕 أكثر من ثلاث مرات يوميا 🌕 مرتين يوميا 🌕 مرة يوميا 🌕 لا شيء مما سبق  **١٠- هل تستخدم أدوات تنظيف بين الأسنان (مثال المسواك، خيط الاسنان اوغسول الفم)؟** لا 🌕 نعم🌕 ١١- **هل تقوم بزيارة عيادة طب الاسنان بشكل اعتيادي (كل ٦ أشهر أو بشكل سنوي)؟** لا🌕 نعم🌕 **إذا الإجابة لا، متى كانت آخر زيارة لعيادة طب الأسنان**؟ **_________________** |
| --- |
