# Supplementary material for: Barriers and facilitators for oral health screening among tobacco users: a mixed-methods study
Source: BMC Oral Health. 2024 Mar 5;24:306. doi: 10.1186/s12903-024-04084-1 (PMC10913556; doi:10.1186/s12903-024-04084-1)
Supplement: Supplementary file 3 — Supplementary Material 3. [file 12903_2024_4084_MOESM3_ESM.docx]

Supplementary File 3. The translated and original responses to prompt questions concerning the facilitators and barriers to oral health screening (n=29).

| **Participant ID (gender/age)** | **Q1: What motivates and helps me to visit the dentist for an oral and dental health screening is …. [original]** | **Q2: I do not visit the dentist for an oral and dental health screening because …. [original]** |
| --- | --- | --- |
| P01  (M, 20) | *When I feel pain, to keep my teeth clean and to keep my mouth smell clean*  *عند الإحساس بالألم، للحفاظ على نظافة اسناني ورائحة فمي* | *Dental visits are not required/lack of dental appointments*  *عدم الحاجة للزيارة، عدم توفر مواعيد* |
| P02  (M, 32) | *When I feel pain*  *عند الاحساس بالألم* | *The lack of time and high costs*  *عدم توفر وقت مناسب، التكلفة العالية* |
| P03  (M, 20) | *When plaque build-up and reasonable costs*  *عند تراكم الجير، الأسعار المعقولة.”* | *Dental visits are not required*  *عدم الحاجة للزيارة* |
| P04  (M, 41) | *When I recognized a problem in my teeth and when I know a good dentist*  *عند ملاحظة مشكلة في اسناني، معرفة عيادة طبيب جيد* | *The lack of appropriate time availability and long waiting times in the clinic*  *عدم توفر وقت مناسب، طول الانتظار في العيادات* |
| P05  *(M, 30)* | *When I feel pain*  *عند الاحساس بالألم* | *To have aesthetic veneers*  *لتركيب عدسات تجميل* |
| P06  (M, 35) | *When I get advice from others*  *عند تلقي النصائح من الاخرين* | *To have aesthetic veneers*  *لتركيب عدسات تجميل* |
| P07  (M, 40) | *Ease of dental visit*  *تسهيل مواعيد زيارة طبيب الأسنان* | *The lack of dental appointments*  *عدم توفر مواعيد* |
| P08  (M, 43) | *When I feel pain*  *عند الاحساس بالألم* | *The loss of hope in the treatment*  *فقدان الامل من العلاج* |
| P09  (M, 31) | *To remove my dental stains*  *لأزاله تصبغات اسناني* | *The lack of time*  *عدم توفر وقت مناسب* |
| P10  (M, 24) | *To maintain good general health*  *للاهتمام بالصحة العامة* | *The lack of dental appointments*  *عدم توفر مواعيد* |
| P11  *(M, 38)* | *To remove my dental stains*  *لإزالة تصبغات اسناني* | *To remove my teeth staining*  *لإزالة تصبغات اسناني* |
| P12  (M, 48) | *Ease of dental visit and the quality of the dental treatment*  *تسهيل مواعيد زيارة طبيب الأسنان، جودة العلاج* | *The lack of time and long waiting time in the dental clinic عدم توفر وقت مناسب، طول الانتظار بالعيادات* |
| P13  (M, 39) | *When I feel pain*  *عند الاحساس بالألم* | *The lack of interest*  *عدم الاهتمام* |
| P14  (M, 36) | *Ease of dental visit and to keep my teeth clean*  *تسهيل مواعيد زيارة طبيب الأسنان، للحفاظ على نظافة اسناني* | *This doesn’t apply to me* [the participant indicated regular dental visits *لا ينطبق علي* [ |
| P15  (M, 22) | *The quality of the dental treatment*  *جودة العلاج* | *Fearing that my dental problems will worsen and embarrassment from the doctor due to my dental condition*  *خوفا من ان تسوء حالة الأسنان لدي، الحرج من الطبيب* |
| P16  (M, 26) | *To maintain good general health*  *للاهتمام بالصحة العامة* | *Fear of pain*  *خوفا من الألم* |
| P17  (M, 27) | *When I recognized a problem in my teeth*  *عند ملاحظة مشكلة في اسناني* | *The lack of dental appointments and fear that my dental problems will worsen*  *عدم توفر مواعيد، خوفا من ان تسوء حالتي* |
| P18  (M, 29) | *Nothing*  لا شيء | *This doesn’t apply to me* [the participant indicated regular dental visits] *لا ينطبق علي* |
| P19  (M, 22) | *When I recognized a problem in my teeth*  *عند ملاحظة مشكلة في اسناني* | *No specific reason*  *لا يوجد سبب معين* |
| P20  (M, 22) | *To maintain good general* health  *للاهتمام بالصحة العامة* | *The lack of time and the interest*  *عدم توفر وقت مناسب، عدم الاهتمام* |
| P21  (M, 22) | *To keep my teeth clean*  للحفاظ على نظافة اسناني | *This doesn’t apply to me* [the participant indicated regular dental visits] *لا ينطبق علي* |
| P22  (M, 24) | *To keep my teeth clean*  للحفاظ على نظافة اسناني | *Dental visits are not required*  *عدم الحاجة للزيارة* |
| P23  (M, 29) | *Availability of transportation/easier arrival to the clinic*  *توفر المواصلات/ سهولة مواعيد زيارة طبيب الأسنان* | *Dental visits are not required, and the lack of time*  *عدم الحاجه للزيارة، عدم توفر وقت مناسب* |
| P24  (M, 30) | *To keep my teeth clean*  للحفاظ على نظافة اسناني | *The lack of dental appointments*  *عدم توفر مواعيد* |
| P25  (M, 24) | *When tartar build-up*  *عند تراكم الجير* | *Cleaning your teeth twice a day is enough*  *تنظيف اسناني مرتين باليوم يكفي* |
| P26  (M, 24) | *When I recognized a problem in my teeth*  *عند ملاحظة مشكلة في اسناني* | *The lack of interest*  *عدم الاهتمام* |
| P27  (M, 25) | *When I feel pain*  *عند الاحساس بالألم* | *Fearing that my dental problems will worsen*  *خوفا من ان تسوء حالتي* |
| P28  (M, 25) | *The beauty of how your teeth look to others and the discount on examination costs*  جمال مظهر الاسنان للأخرين، الخصومات على أسعار الفحص | *The lack of time*  *عدم توفر وقت مناسب* |
| P29  (M, 24) | *To keep my teeth clean*  للحفاظ على نظافة اسناني | *The lack of dental appointments and not knowing a good dentist*  عدم توفر مواعيد، عدم معرفة طبيب جيد |
